# Supplementary material for: Label-free 4D holotomography with depth-adaptive segmentation for quantitative analysis of lipid droplet dynamics in hepatic organoids
Source: Biophotonics Discov. 2026 Sep 30;3(3):035005. doi: 10.1117/1.BIOS.3.3.035005 (PMC13624778; doi:10.1117/1.BIOS.3.3.035005)
Supplement: Supplementary file 1 [file BIOS_003_035005_SD001.pdf]

Supplementary Table S1 Representative refractive indices of major cellular and lipid-related components reported in the literature, compiled from prior QPI/ODT studies. These values were used as qualitative reference points rather than fixed segmentation thresholds.

Component / material	Representative RI	Wavelength	Experimental context
Cytoplasm	1.347 ± 0.002[15]	532 nm	Live hepatocytes, ODT
Nucleus	1.343 – 1.353[34]	540 nm	Isolated nuclei, QPI
Nucleolus	1.349 – 1.372[35]	532 nm	Live cells, 3D-QPI
Mitochondria	1.41 ± 0.01[36]	546 nm	Isolated mitochondria, RM-DIC
Lipid droplet	1.386 ± 0.004[15]	532 nm	Live hepatocytes, ODT
Triolein (Triacylglycerol)	~1.468[37]	589 nm	Pure triolein
Cholesteryl-ester-rich inclusions	1.559 ± 0.0014[38]	Not specified	CE-rich liquid-crystalline inclusions

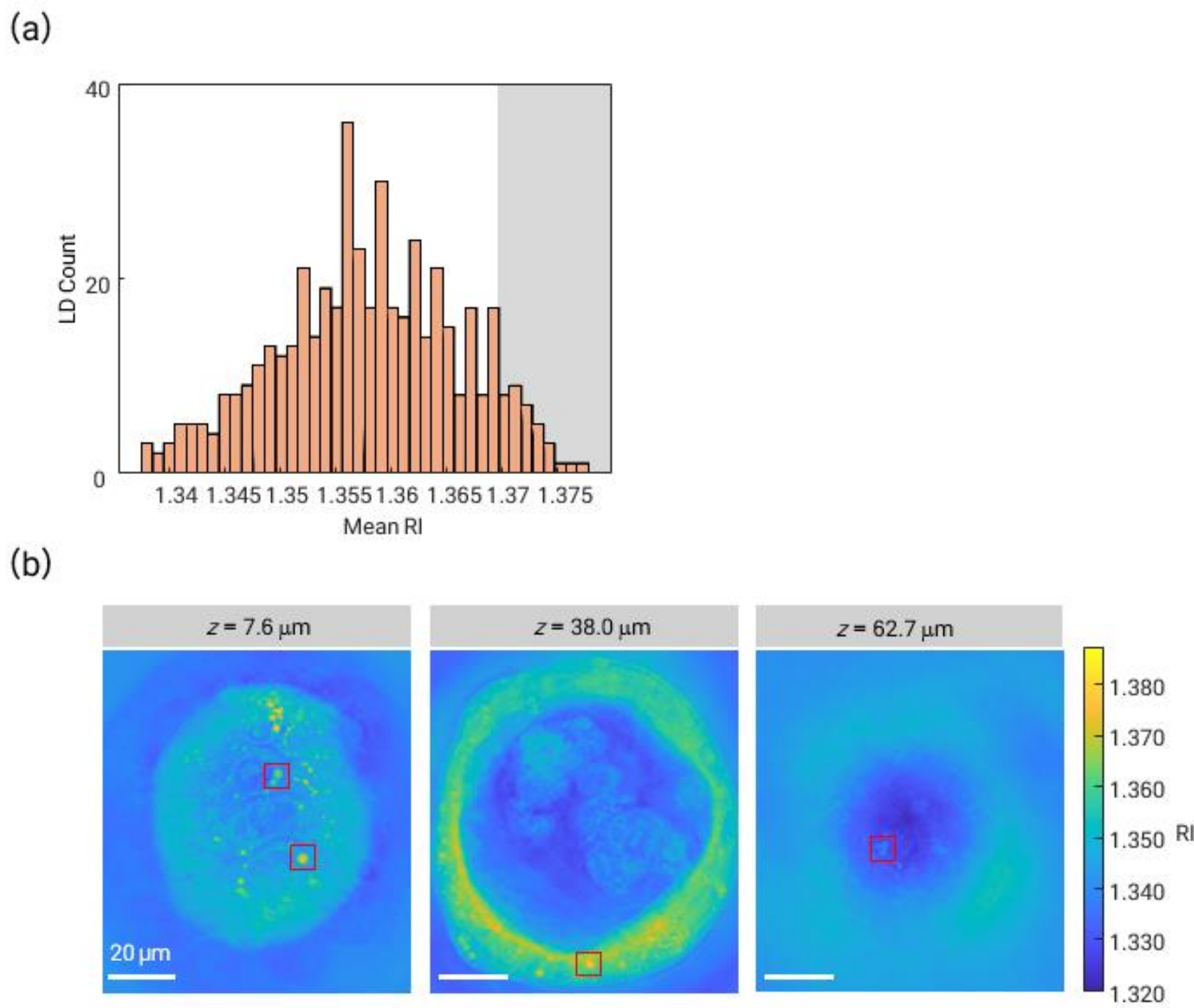

Supplementary Fig. S1 Limitations of conventional single-threshold LD segmentation revealed by RI heterogeneity in 3D organoids. (a) The RI histogram of detected LDs in mHOs revealed a broad distribution of mean RI values, rather than a narrow peak around the commonly used single-cell LD threshold of ~1.37. When applying this fixed threshold (shaded region), only a small subset of high-RI droplets is captured, demonstrating that most LDs in 3D organoids fall below this cutoff and remain undetected. This directly explains why conventional single-threshold segmentation—validated in thin, single-cell conditions—fails in volumetric organoid datasets. (b) Several depth-dependent optical factors inherent to thick organoids further prevent accurate LD extraction using a global RI threshold. First, LDs exhibit z-dependent RI gradients; droplets located at different depths attain their peak RI at different z-slices, producing nonuniform contrast even within the same organoid. Second, intermediate depths contain thick epithelial layers with RI values that exceed those of LDs, causing LD-to-background contrast inversion and leading to substantial under-segmentation. Third, at the topmost *z*-planes, optical aberrations and signal attenuation in thick tissues cause a global reduction in measured RI, further suppressing LD contrast. Together, these depth-dependent variations—LD-intrinsic gradients, epithelial high-RI interference, and depth-related RI attenuation—render global RI thresholding ineffective. These findings highlight the critical need for adaptive, depth-aware thresholding strategies for robust LD detection in volumetric RI tomogram

Supplementary Table S2 Key parameters and rationale for LD segmentation

Parameter	Value Used	Purpose	Expected Effect
RI contrast thresholds, (Δn)	0.005–0.030	Detect LD candidates across depth-dependent variations in RI contrast	Lower thresholds increase sensitivity but may increase false positives; higher thresholds improve specificity but may miss low-contrast LDs
Compactness	< 4.4	Remove irregular high-RI structures	A stricter cutoff removes elongated or irregular objects but may exclude distorted LDs
Solidity	> 0.5	Retain filled, droplet-like objects	A higher cutoff removes fragmented objects but may exclude weakly segmented LDs
Aspect ratio	< 2.0	Exclude elongated structures	A lower cutoff increases selectivity for approximately spherical objects
2D Feret ratio	Max/(min+2) < 1.5	Retain near-circular projected profiles	A stricter cutoff removes elongated projected objects
Size-adaptive refinement	Major axis ≤5px → Δn≥0.010	Suppress small, low-contrast false positives while preserving larger LDs	A higher contrast requirement reduces small false positives but may remove small, low-contrast LDs

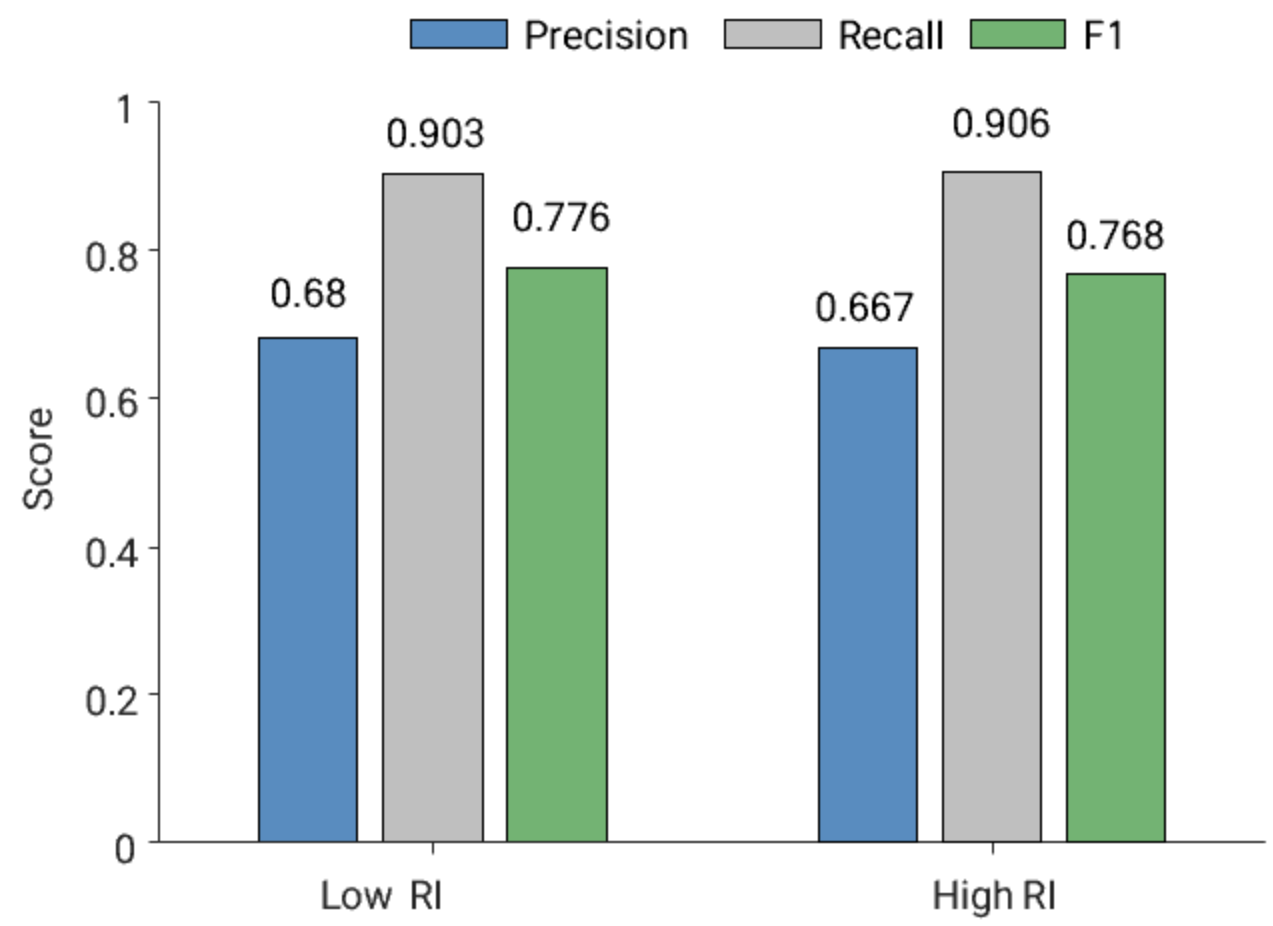

Supplementary Fig. S2 Segmentation performance across RI density strata. Volume-weighted precision, recall, and F1 were comparable between lower- and higher-RI z-plane strata defined by non-LD mean RI (all differences ≤ 0.013).

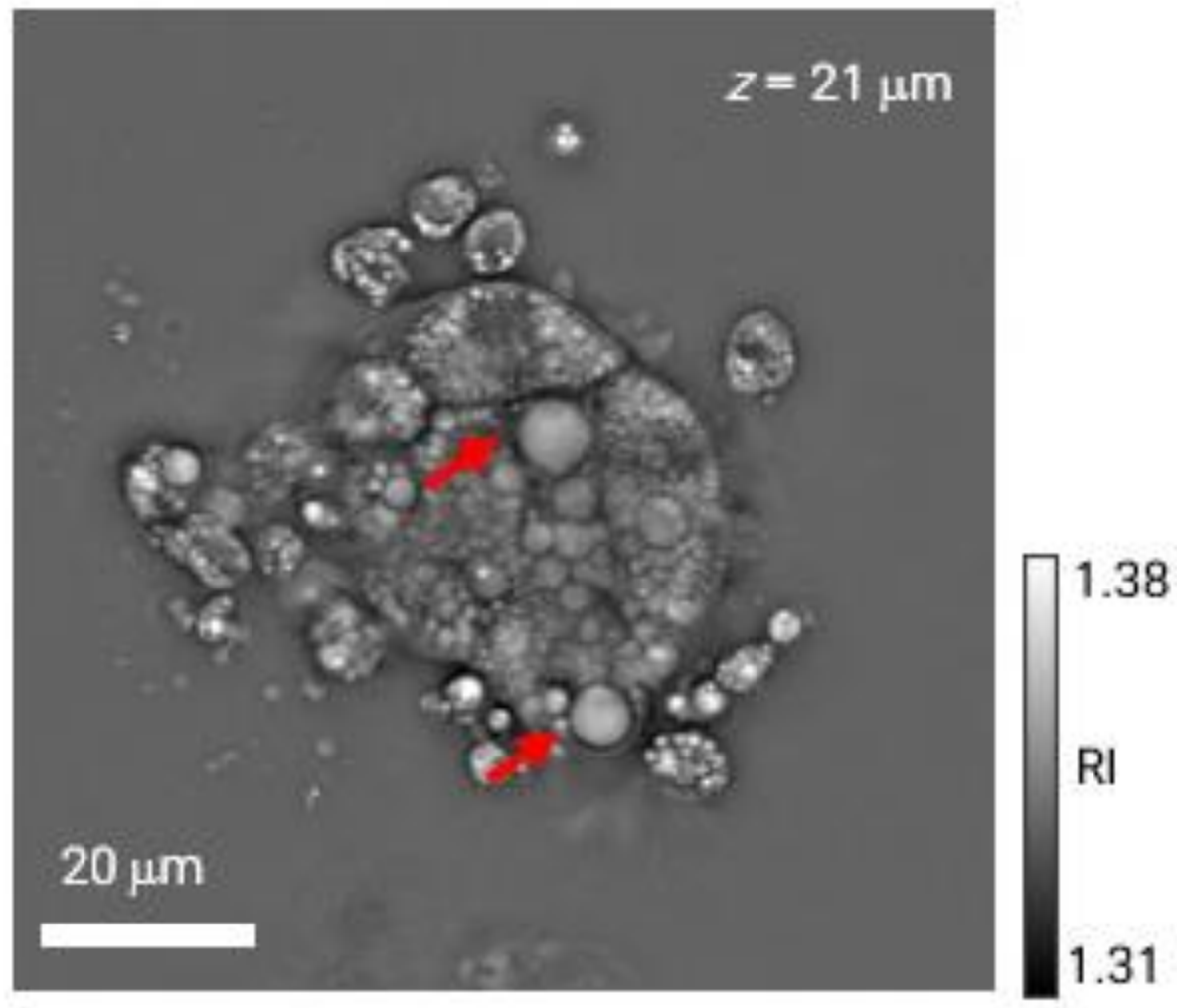

Supplementary Fig. S3 Structural destabilization of mHO under LA treatment. Representative RI slice of a mHO after 24 h treatment with 400 μM LA. The organoid exhibits extensive bulk LD accumulation together with loss of lumen integrity and partial dissociation, indicative of lipotoxic structural deterioration. Red arrows highlight bulk LD accumulation. This organoid represents a severe response phenotype observed in a subset of samples under high dose LA loading. Scale bar, 20 μm.

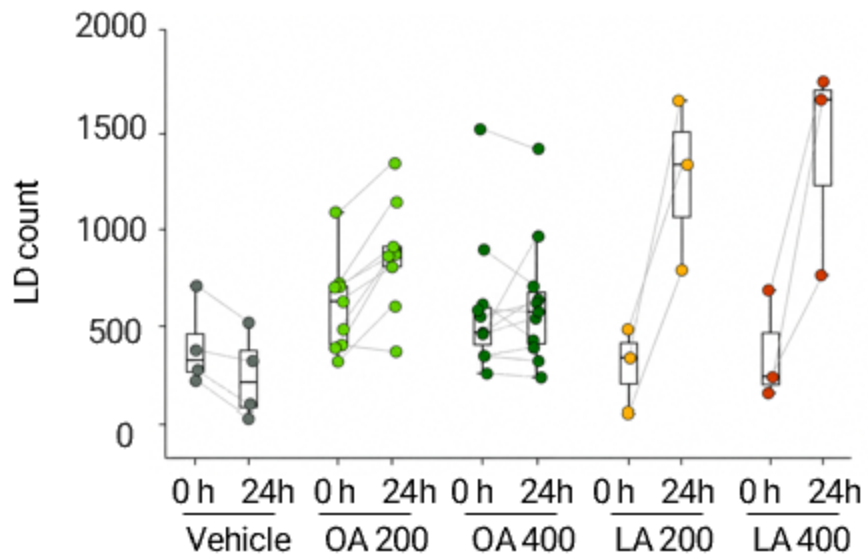

	Vehicle	OA 200	OA 400	LA 200	LA 400
Full Δ	-150	+261	+31.1	+980	+1060
LOO Δ range	[−182, −137]	[+234, +298]	[−16.2, +53.4]	[+798, +1104]	[+818, +1324]

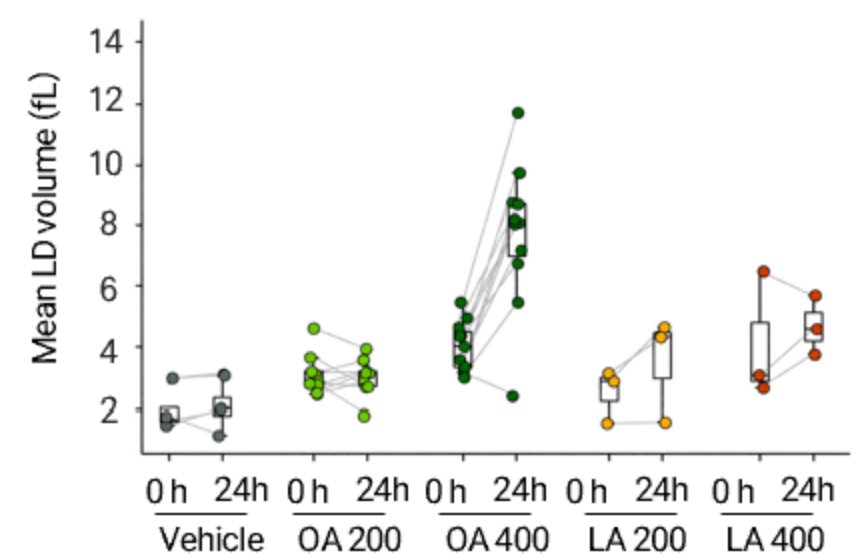

	Vehicle	OA 200	OA 400	LA 200	LA 400
Full Δ	+0.12	-0.10	+3.70	+0.98	+0.60
LOO Δ range	[−0.01, +0.36]	[−0.19, +0.02]	[+3.44, +4.14]	[+0.59, +1.46]	[+0.15, +1.30]

Supplementary Fig. S4 Leave-one-organoid-out robustness of temporal LD phenotypes. Paired 0- and 24-h measurements for each organoid are shown for LD count and mean LD volume across all treatment groups (Vehicle, OA200, OA400, LA200, and LA400). The corresponding tables report the full-sample mean change (Full Δ) and the leave-one-organoid-out range (LOO Δ range), defined as the minimum and maximum group mean changes obtained after excluding each organoid in turn. Major treatment-associated temporal trends were preserved across LOO iterations, whereas effects close to zero showed greater sensitivity to individual organoids. Sample sizes: vehicle *n* = 4, OA 200 µM *n* = 9, OA 400 µM *n* = 11, LA 200 µM *n* = 3, LA 400 µM *n* = 3.

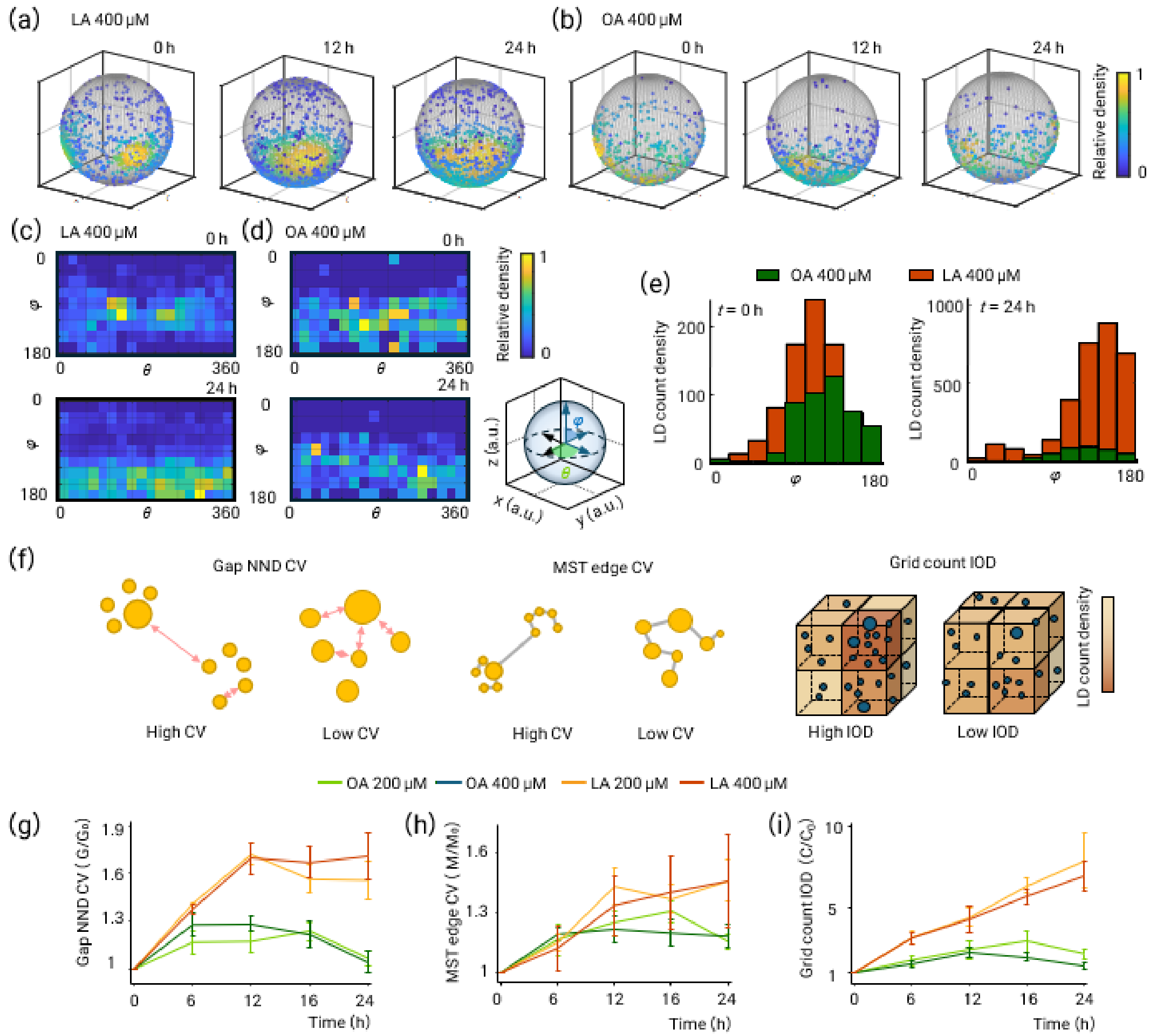

Supplementary Fig. S5 Spatiotemporal mapping and quantification of LD spatial organization within hepatic organoids. (a), (b) Three-dimensional LD centroids were converted to unit direction vectors from the organoid centroid and projected onto a unit sphere, enabling comparison of LD spatial organization independent of organoid size and radial distance. Each point represents an LD direction vector and is color-coded by local angular LD density, defined as the number of neighboring LD direction vectors within a 20° angular neighborhood on the sphere (normalized arbitrary units). (c), (d) Solid-angle–corrected θ–φ LD count density heatmaps at t = 0 h and t = 24 h for LA 400 μM and OA 400 μM, respectively. Solid-angle correction was applied to avoid over-representation near the poles in spherical coordinates. (e) Distributions of LD polar angles (φ) at t = 0 h and t = 24 h, summarizing condition-dependent axial redistribution of LD directions. (f) Schematic overview of three complementary centroid-based geometric metrics that quantify spatial heterogeneity across distinct length scales. (g)–(i) Temporal trajectories of LD spatial heterogeneity metrics across conditions: (g) Gap NND CV (coefficient of variation of nearest-neighbor centroid distances; higher values indicate coexistence of LD-dense neighborhoods and LD-sparse

regions), (h) MST edge CV (CV of minimum spanning tree edge lengths computed from centroid–centroid distances; higher values indicate globally irregular connectivity with short intra-cluster edges and long inter-cluster bridges), and (i) Grid Count IOD (variance-to-mean ratio of LD counts in a 3D grid; values ~1 indicate Poisson-like randomness and values >1 indicate clustering/over-dispersion). Data are shown as mean ± s.e.m. across organoids at each time point. Sample sizes: vehicle $n = 4$, OA 200 μM $n = 9$, OA 400 μM $n = 11$, LA 200 μM $n = 3$, LA 400 μM $n = 3$. Across metrics, LA-treated organoids tended to show increasing spatial heterogeneity over time, whereas OA-treated organoids changed more modestly.

(a)

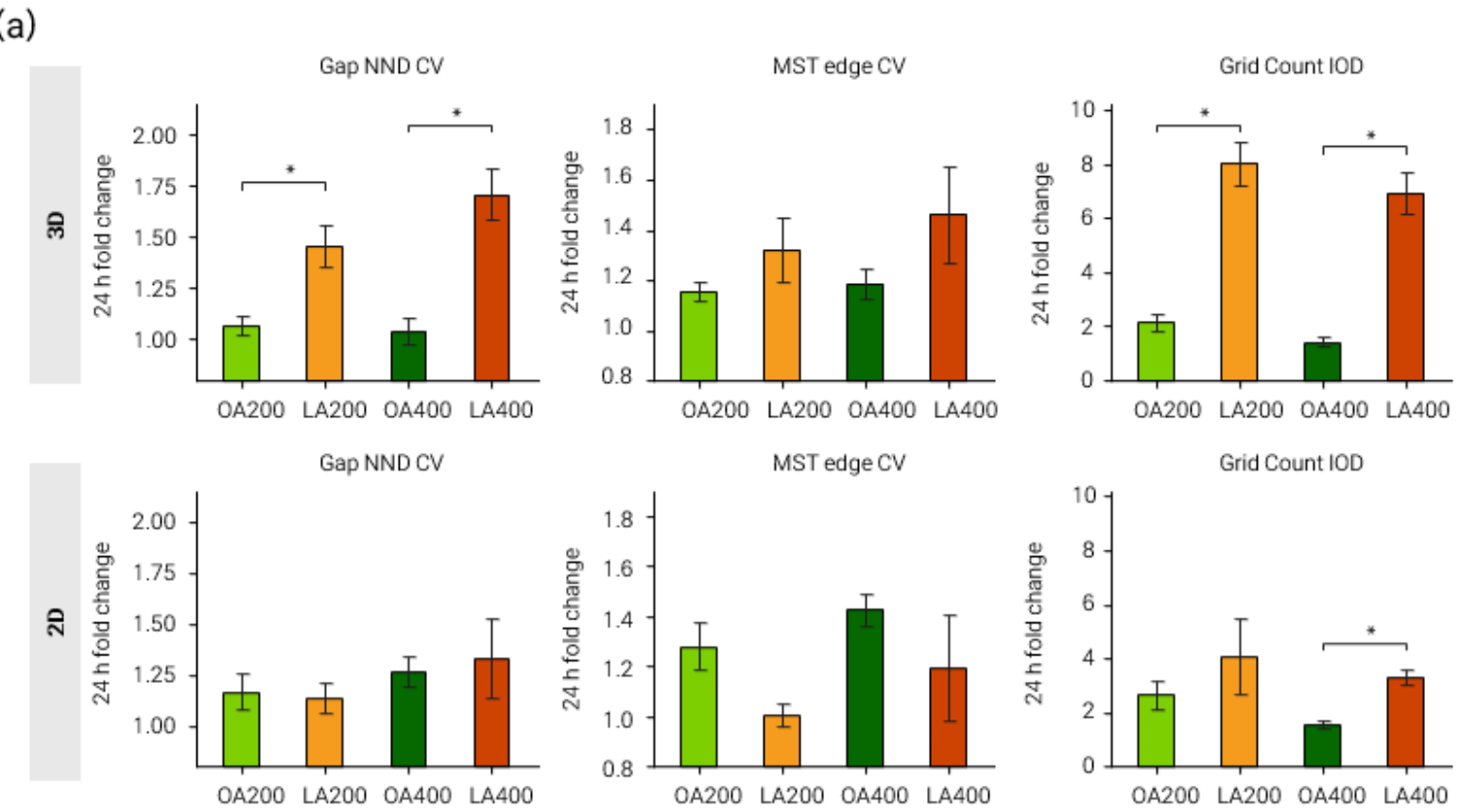

(b)

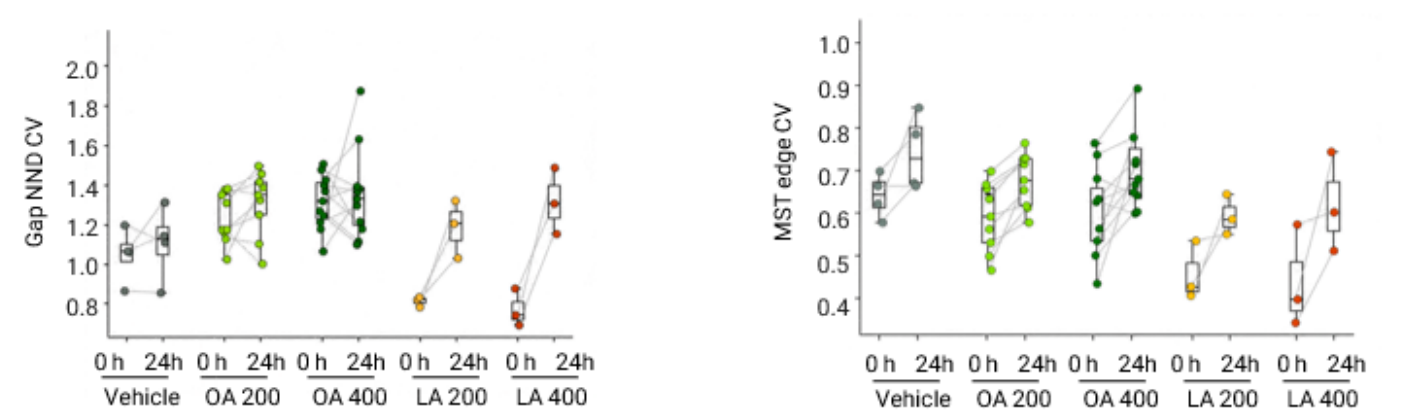

	Vehicle	OA 200	OA 400	LA 200	LA 400
Full Δ	+0.06	+0.08	+0.04	+0.37	+0.54
LOO Δ range	[−0.01, +0.11]	[+0.04, +0.11]	[−0.03, +0.07]	[+0.29, +0.45]	[+0.44, +0.60]

	Vehicle	OA 200	OA 400	LA 200	LA 400
Full Δ	+0.10	+0.08	+0.10	+0.13	+0.18
LOO Δ range	[+0.06, +0.13]	[+0.07, +0.09]	[+0.08, +0.11]	[+0.08, +0.19]	[+0.10, +0.25]

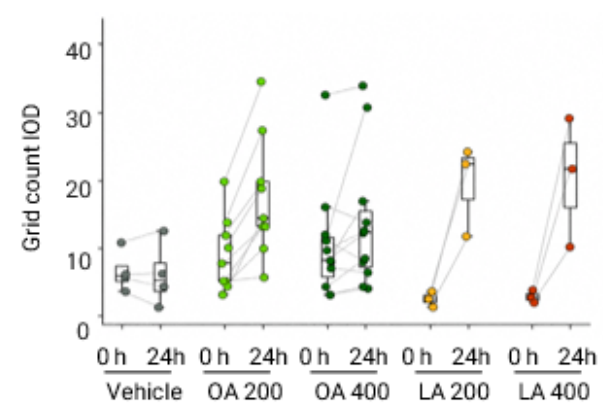

	Vehicle	OA 200	OA 400	LA 200	LA 400
Full Δ	−0.44	+8.44	+3.29	+17.0	+17.6
LOO Δ range	[−1.15, +0.18]	[+7.63, +9.42]	[+1.74, +3.94]	[+14.6, +20.3]	[+13.6, +22.3]

Supplementary Fig. S6 Comparison of 3D and 2D LD spatial remodeling and leave-one-organoid-out robustness. **(a)** Comparison of 24-h fold changes relative to baseline (0 h) in Gap NND CV, MST edge CV, and Grid Count IOD calculated from the original 3D LD coordinates (top) and a 2D MIP-based representation (bottom) for OA- and LA-treated organoids at matched concentrations. Data are shown as mean ± SEM. Asterisks indicate significant OA–LA differences (Mann–Whitney U test, $p < 0.05$); comparisons without asterisks were not statistically significant. OA–LA separation observed in 3D was attenuated after 2D projection for Gap NND CV at both concentrations and for Grid Count IOD at 200 µM. **(b)** Leave-one-organoid-out (LOO) robustness analysis of spatial LD phenotypes. Paired 0- and 24-h measurements for individual organoids are shown for Gap NND CV, MST edge CV, and Grid Count IOD across all treatment groups (Vehicle, OA 200 µM, OA 400 µM, LA 200 µM, and LA 400

μM). The corresponding tables report the full-sample mean change (Full Δ) and LOO Δ range, defined as the minimum and maximum group mean changes obtained after excluding each organoid in turn. Major treatment-associated spatial trends were generally preserved across LOO iterations, whereas small changes close to zero showed greater sensitivity to individual organoids.
